# Nurses' Experiences With Remote Paediatric Patient Asthma Care to Inform Enhanced Implementation: A Descriptive Qualitative Study

**DOI:** 10.1111/jocn.70431

**Published:** 2026-07-07

**Authors:** Souline P. Heil, Laurien Kanis, Lara S. van den Wijngaart, Martinus C. Oppelaar, Maud Heinen

**Affiliations:** ^1^ Department of Nephrology Radboud University Medical Center Nijmegen the Netherlands; ^2^ Department of Surgical Specialties Canisius Wilhelmina Hospital Nijmegen the Netherlands; ^3^ Department of Paediatric Pulmonology Amalia Children's Hospital, Radboud University Medical Center Nijmegen the Netherlands; ^4^ IQ Health Radboud University Medical Center Nijmegen the Netherlands

**Keywords:** Airbridge, eHealth, implementation, leadership, nurse–patient relationship, remote care

## Abstract

**Aim:**

To gain insight into the experiences of nurses in outpatient paediatric asthma care regarding how their profession changed, with an emphasis on workload, nurse–patient relationship, and task shifting, after the implementation of a remote patient care platform (called Airbridge (Luchtbrug)).

**Design:**

A descriptive qualitative study.

**Methods:**

Semi‐structured face‐to‐face interviews were conducted from February 2023 to May 2023, with nurses who were working with Airbridge. Nurses were recruited using a purposive sampling method. An interview guide was developed partly based on the Technology Acceptance Model. Interviews were audio recorded, transcribed, and analysed according to Braun and Clarke's six phases of Thematic Analysis.

**Results:**

Eleven nurses participated in the interviews. Patient centred care and changes of professional practice due to working with remote patient care were identified as the main themes in relation to experiences of nurses after the introduction of a remote patient care platform.

**Conclusion:**

Nurses are pivotal in the successful implementation and sustainability of remote patient care (RPC) platforms, yet they require adequate time and organisational support. As primary users alongside patients, nurses should actively be involved in remote patient care (RPC) practices and take leadership in defining their responsibilities. This study enhances understanding of nurses' experiences with remote patient care (RPC), emphasizing the importance of their role in maintaining the nurse–patient relationship in an advanced professional nursing practice.

**Impact:**

These findings contribute to the understanding of the experience of nurses when working with a remote patient care (RPC) platform regarding effective implementation.

**Reporting Method:**

This study adhered to the COREQ guideline.

**Public Contributions:**

No paper or public contribution.

## Highlights

- Nurses experienced a shortened communication line with their patients, improved insight in patients' health status, and strengthened patient empowerment after the implementation of a remote patient care platform.
- Nurses gained more responsibility and control over their work when using a remote patient care platform.
- The Balance between time and efficiency was challenging due to the shift in tasks after the implementation of a remote patient care platform.
- Nursing leadership is crucial for the successful implementation of remote patient care in nursing practice.

What Does This Paper Contribute to the Wider Global Clinical Community?

- Demonstrates the impact of a remote patient care platform on the professional practice of nurses and patient centred care.
- Highlights How eHealth Influences Nursing Practice and Underscores Adequate Implementation to Ensure Sustainable eHealth Initiatives in Nursing Practice

## 1 Introduction

In recent years, digital health has emerged worldwide as a key area of innovation, reshaping how healthcare is delivered and experienced. Digital health refers to the effective use of technology to improve people's wellbeing with a primary focus on healthcare delivery rather than the technology itself (Fatehi et al. 2020). Digital health includes health services delivered via the Internet and is considered vital for addressing increasing healthcare demands within the constraints of limited resources (Eysenbach 2001; Ross et al. 2016). Within the broader digital health domain, concepts such as eHealth and remote patient care (RPC) have emerged, situated at the intersection of medical informatics, public health, and business.

Research on eHealth implementation has identified several barriers and facilitators and identified the crucial role for nurses in particular (Koivunen and Saranto 2018). However, to enable nurses as facilitators and leaders in the implementation of eHealth in their practice, it is crucial to first create a better understanding of how these interventions are reshaping the nursing profession.

## 2 Background

By leveraging information and communication technologies (ICT), eHealth supports various aspects of healthcare, including diagnostics and follow‐up care (Eysenbach 2001). Through these information and communication technologies, healthcare services can become more accessible, affordable, and personalised (Buntin et al. 2011; Eysenbach 2001). Besides enhancing general healthcare delivery, eHealth plays a crucial role in promoting patient self‐management, which involves patients actively engaging in managing their own health and chronic conditions (Adriaans et al. 2021; Awad et al. 2021; Liu et al. 2022). Digital tools and applications empower patients with greater insight and control over their health by providing feedback and education and stimulate treatment adherence (Thangada et al. 2018), which not only improves quality of life but also helps to reduce the burden on healthcare providers (Awad et al. 2021). Over the past decade, the use and development of eHealth to enhance patient self‐management has expanded significantly, creating new opportunities within healthcare (Penedo et al. 2020). As a result, healthcare systems are becoming more efficient and more responsive to patients' needs and expectations, ultimately advancing the quality and accessibility of care (Elbert et al. 2014).

While eHealth offers many benefits, previous studies have also identified potential drawbacks and barriers to eHealth implementation, limiting its effectiveness in routine practice. Barriers include reduced control over nurses' work processes and technological difficulties (Koivunen and Saranto 2018). On the other hand, facilitators such as digital competencies, positive attitudes towards eHealth, and perceived benefits have been shown to enhance acceptance and facilitation of eHealth implementation (Svendsen et al. 2021). Moreover, healthcare professionals' professional values, personal perceptions, and level of commitment can function as both barriers and facilitators in the adoption and implementation of eHealth interventions (Svendsen et al. 2021). Some studies highlight potential risks associated with eHealth, including healthcare professionals losing a sense of personal connection with patients and being reduced to mere information providers (Nielsen and Mathiassen 2013; Senek et al. 2025; van den Wijngaart et al. 2018), as well as a decline in nurse–patient interaction due to reduced face‐to‐face contact (Oppelaar et al. 2024; Skär and Söderberg 2018). Despite these concerns, when thoughtfully integrated into care, eHealth can significantly enhance clinical decision‐making and strengthen patient‐centred care (Vahdat et al. 2014).

Given these insights, healthcare professionals, and particularly nurses, play a pivotal role in the effective implementation, adoption and acceptance of eHealth technologies (Bail et al. 2021; Koivunen and Saranto 2018). Nurses often serve as the primary contact for patients, facilitating digital self‐management, providing education, ensuring continuity of care, and supporting the integration and effectiveness of eHealth programs through tasks such as outcome monitoring, digital communication, and triaging online queries (Frennert et al. 2023; Hellzén et al. 2022). Their involvement is essential for maximizing the benefits of eHealth by addressing challenges such as patient engagement, technology usability and ensuring the delivery of personalised care. Therefore, nurses' perspectives and involvement need to be fully considered for sustainable implementation of eHealth.

Nevertheless, nurses' experiences and responsibilities with eHealth, integrated into clinical practice, within these systems remain largely underexplored. Particularly in paediatric outpatient care, nurses interact not only with the patients (children) but also, and often primarily, with their parents or carers. Traditionally, paediatric asthma care requires frequent hospital visits, limiting school and after school activities, which often does not meet patient's needs. This dynamic adds an additional dimension to nurses' experiences when working with eHealth programs in this population, compared to working with adults, which is the concern of most existing literature (Tan et al. 2024). Even less is known about nurses' experiences when implementing a paediatric focused eHealth platform. Additionally, eHealth encompasses a wide range of interventions, requiring a deep understanding of varied experiences and perspectives. While a previous study has examined patient experiences with eHealth or the effectiveness of an eHealth intervention related to patient outcomes, prioritizing the user and focusing on feasibility and usability (van der Kamp et al. 2023), even far less is known about the impact of eHealth programs on nurses' perceived workload, nurse–patient relationships, task shifting, and job satisfaction, especially in child care. Gaining insight into these aspects would provide valuable knowledge to support health systems in implementing eHealth programs that not only align with patient‐centred care but also fits efficiently into nurses' daily workflow and responsibilities.

## 3 The Study

### 3.1 Aims

We aimed to gain insight into the experiences of nurses in outpatient paediatric asthma care regarding how their profession changed, with an emphasis on workload, nurse–patient relationship, and shifting of tasks after the implementation of an RPC platform.

## 4 Methods

### 4.1 Design

A descriptive qualitative research design was chosen to gain insight in the perspectives and experiences of nurses working with Airbridge. This design fits the aim to gain a general understanding of the experiences and perspectives of nurses working with an RPC platform (Sandelowski 2000). Semi‐structured individual interviews were conducted by researcher SH.

### 4.2 Study Setting and Recruitment

Pulmonary paediatric nurses, pulmonary paediatric nurse practitioners and pulmonary paediatric nurse consultants working in the field of paediatric asthma were included in this study, hereafter referred to as nurses. Nurses were recruited from all twenty hospitals in the Netherlands that have implemented Airbridge, including both academic and general hospitals. All eligible nurses were invited through e‐mail by researcher MO for participation using purposive sampling. Researcher MO was familiar with the participating nurses but did not have a direct working relationship with these nurses and participation was entirely voluntary. When nurses agreed to participate, their contact information was then forwarded to researcher SH, who was not familiar with the participants prior to the study. Nurses were contacted through e‐mail to schedule an interview. Informed consent was obtained prior to participation.

### 4.3 Inclusion and Exclusion Criteria

Eligibility criteria included: (1) Participants hold a position as pulmonary paediatric nurse practitioner, pulmonary paediatric nurse consultant or pulmonary paediatric nurse practitioner in training and (2) working with Airbridge, and (3) sufficient proficiency of the Dutch language. No additional exclusion criteria were established.

### 4.4 Airbridge (Luchtbrug)

Airbridge is an RPC platform used in routine paediatric asthma care. Included hospitals discuss inclusion in Airbridge with their own paediatric patient population and manage and monitor these independently. The overarching primary goal of Airbridge is improving self‐management, patient outcomes,reducing outpatient visits through digitally available treatment and action plans integrated with monitoring of asthma control, lung function, therapy adherence, and easy patient‐physician contact. Airbridge includes the use of an Asthma Control Test (ACT) to monitor asthma control over time, and it provides patients (and their caregivers) with online digital asthma action and treatment plans based on the ACT. The ACT is a five‐item questionnaire about asthma symptoms indicating whether asthma symptoms are under control or not (Juniper et al. 1999). Users receive feedback on their ACT and lung function scores which refers the patients to their asthma action and treatment plans. Treatment plans include personalised goals established together with the patient as well as general education about prescribed medication and relevant subjects such as lifestyle. Optional features of Airbridge include home spirometry using a portable lung function device and smart inhalers for medication monitoring. Together with the patient is determined which and how often one or more features will be used. Further details on remote monitoring platform can be found in a previous publication (Oppelaar et al. 2025).

### 4.5 Data Collection

Data collection took place from February 2023 to May 2023. Prior to data collection, a topic list was developed in collaboration with the research group SH, MH and MO (supporting information). MH, an expert in qualitative research, provided methodological guidance. The topics perceived usefulness and ease of use were both based on the Technology Acceptance Model (TAM) (Davis and Davis 1989). Other topics were based on pilot interviews conducted by Bachelor of Nursing students in 2022, not included in this study, to gather general information about the subject. The first interviews in the current study served as a pilot interview to evaluate the topic list regarding completeness. No changes in the topic list were necessary after the pilot interview.

Participant characteristics were collected prior to the interview such as age, profession, and work experience. All interviews were conducted in Dutch by researcher SH. Seven interviews took place face‐to‐face; the remaining four interviews were conducted online with Microsoft Teams video connection. Oral consent for audio taping the interview was obtained prior to the start of the interview. Interviews started with an introduction on the goal and background of the study. Audio transcripts were sent to the participants as a form of member check. However, no corrections were received. Interviews were conducted until no new themes emerged during coding, and therefore data saturation was reached as determined collaboratively by the researchers through discussion (SH, MO, MH). There were no repeat interviews necessary. See Table 1 for an overview of the topic list used with example questions.

**TABLE 1:** Topic list Airbridge study with a few example‐questions.

Topic	Example question
Opening	What does your work week currently look like?
Perceived usefulness (TAM)	How do you use Airbridge in your daily work activities? Have any tasks been added or replaced due to the use of Airbridge → Which tasks?
Perceived ease of use (TAM)	What can be changed to improve the use of Airbridge?
Perceived nurse–patient relationship	In what way does Airbridge influence your relationship with the patient? How does Airbridge influence the way you communicate with your patients?
Commitment to nurse–patient relationship	What agreements have you made with your patient regarding Airbridge?

### 4.6 Data Analysis

Braun and Clarke's six‐phase thematic analysis was used, using an inductive reflexive approach 2022. Reflexive thematic analysis (RTA) was chosen for its ability to systematically organise data and facilitate the interpretation of findings. The phases are described in Table 2 for this study. All interviews were conducted in Dutch and for analyses we used the original Dutch transcripts. Quotations, themes and sub‐themes were then translated into English by researcher SH and checked by researchers MO and MH to maintain the original meaning and nuance. The translations were additionally reviewed by a native English speaker to ensure correct spelling and grammar. Data were double‐coded and grouped into themes capturing meaningful patterns, providing an in‐depth understanding of participants' experiences and perspectives by two researchers SH and LK. Data analysis was performed with ATLAS.ti Windows (Version 24.0) (GmbH AtSSD 2024) and took place concurrently during data collection to determine data saturation.

**TABLE 2:** *Six phases of reflexive thematic analysis* (Braun and Clarke 2022).

***Phase 1: Familiarizing with the dataset***
Audio recordings were transcribed verbatim by SH. During both processes, notes regarding analytic ideas or insight were kept, providing an additional check a later phase.
***Phase 2: Coding***
Double coding was conducted independently by researchers SH and LK. Data segments that appear to be interesting, relevant or meaningful regarding the research question were identified by both researchers. Collaborative coding was used to enhance understanding, reflexivity and interpretation (Braun and Clarke 2022), by discussing all codes and deciding on codes using a consensus‐based approach.
***Phase 3: Generating initial themes***
All codes were analysed extensively and discussed by SH and LK. Codes that seem to share a core idea or concept were categorised in code groups.
***Phase 4: Developing and reviewing themes***
In this phase, researchers SH and LK reviewed the code groups until an initial set of candidate themes were identified. All potential themes were then analysed a second time in collaboration with a third researcher (MO). The researchers SH, LK and MO reviewed whether the themes made sense in relation to both the coded extracts and the full dataset. Themes then were renamed, merged or divided into new themes.
***Phase 5: Refining, defining and naming themes***
In this phase, the whole research group reviewed the themes and notes collected in phase 1 once more in relation to the research question and refined the themes. Themes were then translated into English by researcher SH and checked by researchers MO and MH.
***Phase 6: Writing up*** After combining all knowledge and data acquired during the process, results were described and supported by quotes of the participants to answer the research question.

### 4.7 Ethical Considerations

This study was conducted according to the principles of the Helsinki Declaration, version September 2022. The Medical Ethical Research Committee declared that the Medical Research Involving Human Subjects Act (WMO) did not apply for this study (document number: 2023–16,296) Personal information was managed according to the General Data Protection Regulation (GDPR). Audio recordings, transcripts and consent forms were stored in a secured digital network file within the department server. The files are only accessible to MO and SH. Transcript were pseudonymised. Data will be stored for 10 years and subsequently destroyed, in accordance with the EU GDPR.

### 4.8 Rigour and Reflexivity

The research group consisted of researchers SH, LK, MO, LvdW and MH. Both SH and LK are novice researchers and also work as registered nurses in different adult nursing departments not affiliated with this study. Authors MO and LvdW both have a medical background in (digital) paediatric asthma care. Author MH is a senior researcher and expert in qualitative research and supervised the entire research process. To enhance reflexivity researcher MH functioned as an independent senior researcher, leading discussions and asking critical questions throughout the analysis. Reflexivity was further strengthened by ensuring participant confidentiality and by critically reflecting on both the researchers' assumptions and the participants' lived experiences. To enhance the trustworthiness and credibility of the data collection, the criteria of Lincoln & Guba (Lincoln 1985) were used. Credibility was strengthened through investigator triangulation, a rigorous analytic process and by a member check. The dependability of this study was ensured by maintaining an audit trail to document every decision made during the research, along with discussion and consensus‐based independent double coding for all interviews. Two researchers independently double coded and analysed all transcripts (SH, LK). Codes were then compared and discussed until consensus was reached by SH and LK. Hereafter the codes were discussed with MO and supervisor MH. When there was disagreement about a code, a discussion was held until consensus was reached. All themes were discussed and determined through discussion by the research group. In determining the themes and sub‐themes, all researchers engaged in reflective practice, drawing on their prior knowledge and experience to inform the analysis process. COREQ was used to guide transparent and comprehensive reporting (Tong et al. 2007) (suporting informations).

## 5 Findings

### 5.1 Characteristics of Participants

Eleven nurses were interviewed by researcher SH, with an average duration of 30 min and range of 28–45 min. Participants' characteristics are reported in Table 3 below. Interviews were conducted in person at the participants workplace or through an online video connection and were all audio recorded. No nurses dropped out during this study.

**TABLE 3:** Characteristics of participants Airbridge study in 2023.

Age in years	Nurses (*N* = 11)	
Median (IQR)		53 (45.5–56.5)
Female *n (%)*		11 (100)
Hospital *n (%)*		
University		2 (18)
General		9 (81)
Years of work experience in current function, Median (IQR)	10 (2.75–16.25)	
Contract hours, Median (IQR)		27 (17–29.5)
Is Airbridge integrated in EHR *n (%)*		
Yes		2 (18)
No		9 (73)

*Abbreviation: EHR = Electronic Health Record*.

### 5.2 Identified Themes and Subthemes

Two main themes, including ten subthemes, were identified as shown in Table 4. Patient centred care and changes in professional practice due to working with an RPC platform were identified as the main themes in relation to experiences of nurses after the implementation of Airbridge.

**TABLE 4:** *Main themes and sub‐themes Airbridge study*.

Main themes	Sub themes
**Influence on patient centred care**	Shortened communication lines
	Improved insight in patients' health status and timely response
	Mutual trust is essential
	Improved care at the right place
	Strengthened patient empowerment
**Changes of professional practice**	More responsibility and control over work Digital skills are needed
	Balance of time and efficiency is challenging
	Increasing tasks related to RPC

#### 5.2.1 Main Theme: Patient Centred Care

All nurses were asked what they considered to be the definition of patient centred care. Nurses emphasised the importance of patient‐centred care in their work and acknowledged the influence of RPC on this aspect. Nurses generally defined patient centred care as individualised care tailored to each patient and the use of shared decision‐making.

##### 5.2.1.1 Shortened Communication Lines

With the use of RPC, nurses experienced easy and short communication lines with the patients and parents. Sending and receiving messages was perceived as an easy and low‐threshold communication method for both nurses and patients. Nurses mentioned that patients appreciated being able to communicate easily and directly at their convenience, without needing to make a phone call to the nurse or physician. However, one nurse expressed concern that sending messages using the Airbridge inbox might become overly accessible to patients, which could predispose to miscommunication and overuse.

Difficulties mentioned were identification of the specific patient when no profile photo had been uploaded, interpreting text messages without nonverbal cues, and assessing the severity of physical complaints based on text messages alone. Most nurses emphasised that remote care could not replace in‐person consultations fully. Because of the short communication lines with the patient, changes to treatment plan could also be communicated easily and are directly visible for the patients.

Yes, you must check carefully how you write something[…] last time I had a situation with a parent where I thought ‘it seems like they are angry’. So, I called the mother it was just her way of writing something. So yes, you must be careful because you know that writing something could come across as something different than just speech (R8)

##### 5.2.1.2 Improved Insight in Patients' Health Status and Timely Response

With the introduction of RPC, nurses mentioned improved insight into the patient's medication use, therapy adherence, and general health. Through RPC, nurses also gained a better view on the effectiveness of the treatment plan and the patient's overall health status over time. The ACT score, indicating whether asthma is controlled or uncontrolled, also improved nurses' as well as patients' awareness of possible triggering factors for exacerbation, subsequently facilitating better personalisation of treatment plans.

Especially timely response and intervention were frequently mentioned as a major improvement associated with the use of RPC. Mainly using the ACT score, nearly all nurses reported that they could monitor the patients' health better and could therefore anticipate more quickly and accurately in their treatment, potentially preventing pulmonary exacerbations.

You can anticipate quicker than when you must wait until their appointment here in the hospital. Because when something is wrong, not all parents notify you, they wait until they have an appointment. And definitely with such a red score you can directly give advice, so you can actually prevent earlier that they deteriorate further (R9)

##### 5.2.1.3 Mutual Trust Is Essential

Nurses mentioned that consistent use of the RPC platform by patients and parents was required for beneficial use, and this was considered the primary responsibility of patients and parents themselves. Most nurses mentioned that RPC is not suitable for every patient, highlighting the importance of assessing individual patient needs and capabilities before introduction. The introduction of RPC to a patient is often first discussed within the team, but can also be initiated by an individual nurse, and discussed with the patient. However, some nurses reported that they invested time and effort, and this effort was not always reinvested by the patient. All nurses educated their patients about the importance of calling the hospital when any acute health issues arise instead of relying solely on a response in the email inbox. This required trust from nurses to their patients. Similarly, patients needed to trust nurses to provide timely and appropriate responses to their questions. Therefore, establishing a strong patient relationship was mentioned as a requirement prior to the introduction of an RPC platform.

It depends, there are some children and parents I trust enough to notify me when something is wrong […] But if there are patients I worry about, and when I cannot find more information, then I call them to check if they are alright. (R4)

##### 5.2.1.4 Improved Care at the Right Place

Some nurses described RPC as a way of keeping up with the current digital era. One nurse also mentioned that with the COVID‐19 pandemic, digital communication became more normalised and has shifted care from hospitals to the home environment of patients, leading to a reduction in hospital visits. Nurses observed that this shift towards digital care is generally appreciated by most patients. However, several nurses also mentioned that RPC cannot replace in‐person care entirely. Seeing patients in real life remained essential, especially for those with unstable conditions, while patients with stable health could be monitored remotely and seen less frequently. Due to this shift, nurses also noted that the outpatient population attending the hospital is gradually becoming more complex.

You can organise differently, because now you have more time for other tasks or patient categories that require more complex care. So, you can arrange tasks differently. And of course, you want to provide care where it is most needed […] and that has much value, you want to serve everyone (R10)

I have to say, we see children who are stable less often, so if we see the children in person, those are often more ill, and more things need to happen. (R8)

##### 5.2.1.5 Strengthened Patient Empowerment

With the use of RPC, nurses experienced those patients becoming more empowered in managing their own condition and treatment. Most nurses highlighted how RPC increased patients' and their parents' awareness and understanding of their condition through regular reminders for symptom questionnaires, personalised information in the treatment plan, better appraisal of symptoms with ACT or lung function outcomes, thereby increasing the self‐management of the patients. RPC also provides an overview for patients and parents to monitor the patients' health and physical complaints over time. This overview helps to discuss the patients' health and care plan during outpatient consultations.

Patients have easy access to contact your team. So, you create some self‐management of patients and awareness because they can see that the lung function score is lower, what do I need to do, do I have more physical complaints, can I discuss this. Or the ACT is lower, what can I do myself, when do I need help […] (R10)

It also creates awareness in patients. They can anticipate and learn to recognise, ‘Okay yes we have a question, hay fever season is coming soon, I think we will start medication already’. That is very good, because if Airbridge hadn't been there, then they would have waited until the next consultation. And then all those hay fever symptoms would have gotten out of hand. People wait for approval of a doctor or nurse, but through communicating with Airbridge people really gain insight and take control themselves (R9)

#### 5.2.2 Main Theme: Changes of Professional Practice

With the introduction of RPC, changes were experienced in the professional practice of nurses.

##### 5.2.2.1 More Responsibility and Control Over Work

RPC was implemented differently across organisations, and approaches taken by nurses varied. Several key requirements for successful integration of remote care into practice were identified, including sufficient time, adequate workspace, and team support.

Almost all nurses mentioned a lack of clearly defined roles and responsibilities related to RPC. For example, it was often unclear who was responsible for responding to messages in the inbox and how quickly replies were expected from a team member and by whom. Additionally, the roles and responsibilities of physicians varied across organisations and were often unclear. Therefore, the responsibility for managing the RPC platform often fell primarily on the nurses covering most of the tasks. Often, the nurse would also update the RPC platform on behalf of the physician and respond to the emails in the inbox. In some hospitals, the doctor's assistant would be responsible for the inbox. However, sometimes nurses still felt responsible, wanting to ensure that nothing would be overlooked.

Some nurses only accessed the RPC platform during designated (outpatient clinic) hours, while others were working in clinical departments checking their inbox during those shifts. Nearly all nurses reported managing tasks between other responsibilities whenever time allowed. When overall workload increased, RPC tended to get a lower priority, leading to longer response times.

Response times to the inbox differed between nurses, generally ranging from two to three working days. Most nurses appreciated the flexibility of responding at their own pace, as it reduced the number of phone calls they received during work and allowed them to reply when it best fitted their schedule. This was often mentioned by nurses as a huge benefit of RPC. However, some nurses mentioned feelings of pressure regarding response times, because they did not want patients to wait a full week for a reply. As a result, some nurses indicated responding to RPC messages in their own spare time. One nurse even felt the need to reserve time during her outpatient clinic hours for an additional digital check‐up on patients, because patients were visiting the hospital less frequently. Most nurses expressed a desire to check the inbox as often as possible to stay on top of patient needs, fearing that they might miss important information.

I work together with a colleague, and we sort of discusses that I would cover Airbridge on Monday and Tuesday. And she would cover Airbridge on Wednesday, Thursday and Friday. When I have weekend shift or night shift, I also check if there are messages in the inbox (R2)

[…] Because I have a small contract, it gives me the feeling of being there for the patients. Because I would think that two days a week is not enough availability. And because of Airbridge, I don't need to be available by phone, I can just do something different with my kids and when I have spare time, I will check Airbridge. And this gives me more satisfaction, being available despite having a small contract (R4)

##### 5.2.2.2 Digital Skills are Needed

To work effectively with remote care, nurses needed to develop digital skills. These skills were often self‐taught and passed on to other colleagues. Although an instruction manual was available, nurses often relied on experimental learning and figured things out by themselves. Most nurses indicated that the use of remote care required them to be more technically proficient to use the platform with ease.

Nurses also mentioned various technical difficulties when working with remote care, like connection issues with remote care tools, such as the portable spirometer or smart inhaler. These difficulties led to a reduced usage of tools, decreased patient motivation, a loss of time and increased complexity of their work. Despite having the option to contact the platform developers, nurses often attempted to resolve these issues themselves.

The user friendliness could be improve more. So, the connection to the EHR and portable spirometer could become more user friendly (R5)

##### 5.2.2.3 Balance of Time and Efficiency Is Challenging

Most nurses experienced an increase in workload due to use of RPC. The increase in workload was most noticeable directly during the implementation of RPC. Nurses noted that using RPC effectively required an initial large investment of time. However, not all nurses experienced the added work tasks as a burden. Some nurses noted a reduction in workload, as Airbridge led to fewer phone calls from patients during work hours, resulting in fewer interruptions and improved focus on other tasks.

In most cases, RPC was not integrated with the hospital's electronic health records (EHR). This lack of integration led to double documentation of medication and treatment plans, resulting in extra administrative work for nurses. Some nurses feared that this could lead to safety issues in care. As a result, nurses felt the need to continuously check whether the content on the EHR as well as the RPC platform were in concordance. In hospitals where integration was available, nurses recognised the value of integration of the RPC platform with the hospital's EHR system and emphasised its importance for efficient use.

Yes, it depends. Sometimes it is high, but it also depends on how busy the day is (R6)

But it also saves time. When children use Airbridge, and when they send an inbox message. I can check with the physician what to reply. And I can send them a reply with advice through the inbox and check if they have read the message. That way I don't always need to call so this saves times (R2)

##### 5.2.2.4 Increasing Tasks Related to RPC

With the introduction of RPC the work tasks and responsibilities of the nurses changed significantly. One of the main changes was the responsibility of monitoring the inbox. All nurses reported that additional actions were required after receiving an inbox message from a patient or when a patient reported a low ACT score. These actions included calling the patient, replying via the platform, or consulting with a colleague or physician. Additional tasks were managing the inbox and ACT scores. RPC also required several administrative tasks. Nurses were responsible for registering new patients, tracking all enrolled patients, and checking whether patients remained active on the platform. Additionally, RPC influences outpatient consultation where nurses had to make agreements with patients about the frequency of ACT completion, treatment goals, and discuss patient motivation. They also needed to provide instructions about using Airbridge tools and the platform in general.

If you already know that a patient has a low ACT, then you can move an appointment forward or call to discuss things for example inhalation techniques […] (R4)

## 6 Discussion

This study aimed to gain insight into the experiences of nurses in outpatient paediatric asthma care regarding changes in their profession after implementation of a remote patient care (RPC) platform, with an emphasis on workload, nurse–patient relationship and task shifting. Patient‐centred care and changes in professional practice were identified as two overarching themes in nurses' experiences with RPC. Overall, nurses experienced the implementation of RPC as beneficial for patient care, while simultaneously introducing new challenges. RPC was implemented in complex and dynamic environments and requires adequate implementation strategies to ensure long‐term integration and consistent use in clinical practice.

Regarding patient centred care, nurses' insight into the actual health status of their patients improved because of RPC and the ability to monitor patients at home which made it possible to intervene at an early stage. A systematic review of Tan et al. (Tan et al. 2024) showed that adult patients using RPC also gain more insight into their own condition themselves and that self‐efficacy and therapy adherence are stimulated by increasing patients' knowledge, skills and positive behaviours (Thomas et al. 2021). Similar, promising results on medication adherence and asthma‐related outcomes were seen as a result of eHealth interventions for asthma management in paediatric patients (van der Kamp et al. 2023). In addition, such interventions may empower patients to manage their condition more effectively, thereby enhancing the nurse–patient relationship and increasing patient participation in shared‐decision making (Senek et al. 2025). However, the effectiveness also depends on a frequent and adequate use of the RPC platform, which constitutes a key responsibility for patients and their parents or carers, as highlighted by our study findings. Therefore, establishing a strong foundation of trust within the individual care relationship is essential prior to the introduction and use of RPC (Senek et al. 2025). At the same time, RPC also poses risks because communication is often asynchronous which can lead to misunderstandings (Lie et al. 2019; Oppelaar et al. 2024). Furthermore, relying on digital communication in writing without both verbal and non‐verbal cues demand different skills and may also challenge the process of forming an essential caring nurse patient relationship (Lie et al. 2019). Therefore, outpatient clinics cannot be entirely replaced by RPC; face‐to‐face contact remains essential for the development of a caring nurse‐patient relationship (Lie et al. 2019). In our study, the importance of establishing a caring nurse–patient relationship before introducing a RPC platform became even more apparent. Paediatric patient care inherently involves parents or carers and patients are still in a continuous process of growth and development. As a result, building this relationship may require a more tailored approach. One example, suggested by nurses in our study, is the frequent updating of the patient profile within the RPM platform to help maintain a personal connection. Nevertheless, nurses perceived asynchronous communication as beneficial, as it required less time than contacting patients by telephone. These findings are similar to a previous study, which suggests that nurses find working on direct patients' care, through asynchronous communication, less demanding in contrast to telephone consultation (Frennert et al. 2023).

Regarding the change in profession, the introduction of RPC impacted both patient care and the balance in responsibilities between nurses and physicians. Similar to the findings of (Lottonen et al. 2024), the introduction of RPC led to increased task shifting from physicians to nurses where nurses received more tasks and responsibilities that were previously allocated to physicians. Consequently, we found that nurses strongly felt they had the primary responsibility for the use and supervision of the RPC platform whereas they experienced little involvement from physicians. This strong sense of responsibility, which is a common trait in professional nursing (Jia and Yue 2025), resulted in some cases in an increase in workload and feelings of pressure to react on time. This is in line with Frennert et al. (Davis and Davis 1989) who discussed that the use of eHealth leads to more invisible labour for nurses as they performed the bulk of the tasks in the management of the RPC platform and the responsibilities which came with it. For example, taking care of the eHealth application and the data retrieved from the application creates different work routines and the need for different skills, which is not always recognised by organisations and for which nurses do not receive dedicated time (Frennert et al. 2023; Lottonen et al. 2024). In our study, nurses needed a wide variety of digital skills to handle the RPC platform. The need for nurses digital skills is also highlighted by findings from Frennert et al. (2023) who adds that patients often first contact nurses for technical support when there are issues with the application which suggest that nurses also need problem solving digital skills. Adequate training and support may improve long‐term program maintenance according to the implementation and sustainability infrastructure domain of the PRISMs framework (Feldstein and Glasgow 2008).

Although task shifting is a conscious and necessary strategy, these findings do emphasise the continued need for establishing clear and shared agreements about the management and responsibilities in working with new RPC interventions. We found that this strongly requires leadership of nurses in the implementation of these new tasks and responsibilities and leadership of physicians to support and oversee these changes (Bail et al. 2021; Ronquillo et al. 2023). Previous studies have illustrated that this leadership, which includes innovative, visionary and supportive behaviour, is highly relevant to create a supportive culture that improves nurses' intention to use and use behaviour of digital health interventions (Laukka et al. 2022; Ronquillo et al. 2023). Our results highlight the importance of nursing leadership on the successfulness of the implementation of e‐health in nursing practice.

This study identified several strategies for adequate RPC implementation into daily practice, such as training about RPC platform use for nurses and patients. As well as organisational aspects, such as time and support, which could increase a positive attitude among the nurses (van den Wijngaart et al. 2018), and as a result increase sustainable RPC platform use. This builds on the findings of Cremers et al. (Cremers et al. 2021), who state that training, acceptance and organisational factors are determinants when implementing RPC. Lack of interoperability of the RPC platform into the EHR resulted in less efficient workflow and posed a potential risk for clinical errors (Frennert et al. 2023). Our findings add to previous research where good interoperability is regarded as essential for successful and sustainable implementation (Ross et al. 2016; van den Wijngaart et al. 2018).

### 6.1 Limitations and Strengths

This study has limitations, such as generalisability in terms of sample variation. Only female nurses were included, although this is reflective of the Dutch nursing profession. We expect that this aspect has limited influence on how the nurses perceived their experience with RPC. During the recruitment, the risk of selection bias could not be fully excluded, as only nurses with prior experience using the RPC platform were included. It is possible that this has created a positive bias towards the intervention and implementation of Airbridge. However, nurses with experience were intentionally selected in order to provide informed and meaningful reflection. To minimise further selection bias, no additional restrictive inclusion criteria were applied. Moreover, the informed consent procedure explicitly stated that all data would be anonymised and that the study aimed to explore participants' experiences, enabling respondents to speak freely about their views. The transferability of our findings is strengthened by providing a detailed description of the context in which the RPC platform was used, in addition to the description of study settings and participant characteristics. To maximize generalisability and variation, nurses from both academic and general hospitals were included. To limit potential bias during the research process, a pilot interview was conducted to practice interviewing and evaluate the topic list. Interviews were also conducted by one researcher, which allowed the researcher to use experience gained in interviews in the following interviews.

Strengths of this study are its explorative nature where ‘real‐world evidence’ was collected (Cremers et al. 2021). In addition, participants were nurses with plenty of experience with the use of RPC and they monitored a great variety of patients. To enhance the trustworthiness of this study, various actions were taken such as having two researchers analyse the data independently and consulting an expert in qualitative research (MH).

### 6.2 Implications and Recommendation for Further Research

For further research, it would be valuable to use an implementation framework to examine whether nurses would benefit from a more efficient and structured implementation and use of RPC, such as the PRISMs framework, to ensure the safe use of RPC platforms and safeguard the workload of nurses. In addition, we would recommend further identifying the implementation of eHealth, such as RPC, in the paediatric care specific as this potentially requires different implementation strategies. Matching implementation strategies to address hindering factors is needed for an adequate implementation of eHealth into the healthcare system (Feldstein and Glasgow 2008). One of these strategies is good communication between RPC platform developers and end‐users, like nurses, about the developments of RPC platforms and educating nurses about the use of RPC platforms. With this knowledge, organisations or the RPC platform developers must recognise and acknowledge the impact of eHealth on the work of nurses as an important group of end‐users by involving nurses in the development and implementation of an RPC platform.

## 7 Conclusion

These findings contribute to the understanding of the experience of nurses when working with a remote patient care (RPC) platform regarding the nurse–patient relationship and impact on their profession. The use of an RPC platform resulted in shortened communication lines between nurses and patients, although challenges emerged due to the nature of digital communication. Therefore, maintaining a solid nurse–patient relationship in the use of RPC platforms is essential. Nurses and patients are the primary users of the RPC platform and should therefore play a substantial role in the implementation and sustainability of the RPC platform. This descriptive qualitative study has increased our knowledge about nurses working with RPC and highlights that nurses should take their role and show leadership in defining their responsibilities in working with RPC.

## Author Contributions

Souline P. Heil: conceptualisation, writing original draft, data collection and analysis. Laurien Kanis: Data analysis and review. Lara S. van den Wijngaart: Review. Martinus C. Oppelaar: Conceptualisation, data analysis and review. Maud Heinen: Conceptualisation, Review.

## Funding

The authors have nothing to report.

## Ethics Statement

Ethical approval was obtained from the Medical Ethical commission Oost‐Nederland.

## Conflicts of Interest

The authors declare no conflicts of interest.

## Supporting information


**Data S1:** Supporting Information.

**Data S2:** Supporting Information.

## Data Availability

The data that support the findings of this study are available from the corresponding author upon reasonable request.
