# Supplementary material for: Nurses' Experiences With Remote Paediatric Patient Asthma Care to Inform Enhanced Implementation: A Descriptive Qualitative Study
Source: J Clin Nurs. 2026 Jul 7;35(11):5073–83. doi: 10.1111/jocn.70431 (PMC13629255; doi:10.1111/jocn.70431)
Supplement: Supplementary file 1 [file JOCN-35-5073-s001.docx]

# Interview guide and topic list Dutch

| Interview guide (Dutch) | | |
| --- | --- | --- |
| Topic | **Vragen** | **Vervolg vraag + opmerking interviewer** |
| Introductie | Voorstellen + doel van het onderzoek  *Welkom, mijn naam is x en ik ben bezig met mijn studie verplegingswetenschap. Voor mijn afstudeeronderzoek ben ik nieuwsgierig naar de ervaringen van verpleegkundigen die werken met de Luchtbrug. De interviews zullen een code krijgen waardoor gegevens niet te herleiden zijn naar personen. Alleen de onderzoekers weten welk interview bij wie hoort.*  Tijdsduur:  *Het interview zal ongeveer 30 tot maximaal 45 minuten duren. Ik wil graag het interview opnemen als u dit goed vindt. Hoe mag ik u aanspreken?*  *Heeft u verder nog vragen vooraf?* |  |
|  | Audio starten  *Dan beginnen we nu met het interview.* |  |
| Topics Toepassen luchtbrug | | |
| Begin vraag | Hoe ziet uw werk week er op het moment uit? |  |
| Ervaren bruikbaarheid | | |
| werkzaamheden | Hoe past u Luchtbrug toe in uw dagelijkse werkzaamheden?  Hoe zijn uw werkzaamheden als verpleegkundige (specialist/consulent) veranderd sinds u Luchtbrug gebruikt?  Hoe zagen uw werkzaamheden er eerst uit zonder het gebruik van Luchtbrug?  Zijn er taken bijgekomen door Luchtbrug of zijn er taken vervangen door het gebruik van Luchtbrug?--> Welke taken zijn dit?  Welke afspraken zijn er gemaakt binnen het team met betrekking tot het bijhouden van Luchtbrug? |  |
| Effectiviteit | Heeft u het idee dat uw werkzaamheden makkelijker zijn geworden of juist ingewikkelder sinds het gebruik van Luchtbrug? Kunt u dat toelichten? |  |
| Topics Ervaren gebruik | | |
| Gebruik | Wat zou naar uw mening nog verandert moeten worden het gebruik van Luchtbrug (nog meer) verbeteren?  Zou u Luchtbrug aanbevelen aan een collega van een ander ziekenhuis die nog niet met Luchtbrug werkt?  Kunt u een cijfer geven op een schaal van 0-10? (0 staat voor ‘zeker niet’, een 10 voor ‘zeker wel’)?  Kunt u aangeven waarom het geen xx (hoger cijfer) is?  Kunt u aangeven waarom het geen xx (lager cijfer) is? |  |
| Tools/appplicaties? | Welke tools van luchtbrug gebruikt u in uw dagelijkse werkzaamheden?  Hoe ervaart u het gebruik van deze tools?  Wat belemmert jullie om deze tools te gebruiken? |  |
| Topics Vormgeven verpleegkundige-patiënt relatie | | |
|  | Op welke manier beïnvloedt Luchtbrug uw relatie met de patiënt? | Hoe ervaart u dit?  Ervaart u hier beperkingen in? |
| Persoonsgerichtezorg | Hoe beïnvloedt Luchtbrug de manier van communiceren met uw patiënt? | Hoe ervaart u dit? |
|  | Graag wil ik het met u hebben over de begrippen persoonsgerichte zorg en eigen regie in relatie tot Luchtbrug.  Kunt u mij vertellen wat in uw ogen persoonsgerichte zorg is?  Op welke manier draagt Luchtbrug hierbij aan?  Op welke manier draagt Luchtbrug bij aan de eigen regie van de patiënten. Kunt u daar iets over vertellen?  Op welke manier draagt Luchtbrug bij aan het proces van Samen Beslissen met patiënten? Kunt u daar iets meer over vertellen? |  |
| Topics Toewijding aan verpleegkundige-patiënt relatie | | |
|  | Afspraken met uw patiënten.  Welke afspraken heeft u met uw patiënt gemaakt met betrekking tot Luchtbrug?  Heeft u situaties gehad waarin deze afspraken niet nagekomen konden worden?  Zijn er situaties waarin uw twijfelt wat u moet doen in relatie tot het werken met Luchtbrug?  14d. Hoe gaat u er mee om als een patiënt zich niet houdt aan de gemaakte afspraken? |  |
| Slot vraag | Toegevoegde waarde  Vindt u dat Luchtbrug een toegevoegde waarde heeft op uw werkzaamheden als verpleegkundige?  Kunt u daar iets meer over vertellen? Wat is de toegevoegde waarde van Luchtbrug voor uw werkzaamheden? | Wat is de toegevoegde waarde van Luchtbrug op uw werkzaamheden als verpleegkundige. |
|  | Zijn er nog andere dingen/zaken die u wil benoemen in relatie tot het werken met Luchtbrug of naar aanleiding van dit interview? |  |
| Afsluiting | Bedankt voor uw deelname aan dit interview.  Hoe verder:  Indien u het interessant vindt kan ik u een verslag van het onderzoek opsturen. Heeft uw daar interesse in? Ja/Nee  Ik zou u graag het transcript van dit interview willen opsturen zodat u dit kunt doorlezen. Mocht u dan nog aanvullingen hebben dan kunt u dit aan mij doorgeven. Het is niet verplicht om te reageren. Vindt u dat goed? Ja/Nee  Zo ja, naar wel (mail)adres mag ik het verslag en/of transcript sturen? |  |

# Interview guide and topic list English

Table 1. Topic list individual interviews

| Interview guide (English | | |
| --- | --- | --- |
| Topic | **Questions** | **Follow-up question + interviewer’s remark** |
| Introduction | **Introduction + purpose of the study**  Welcome, my name is x and I am currently studying nursing science. For my graduation research, I am interested in the experiences of nurses who work with Airbridge The interviews will be coded so that the data cannot be traced back to individuals. Only the researchers, will know which interview belongs to which participant.  **Duration:** The interview will take approximately 30 to a maximum of 45 minutes. I would like to audio-record the interview if you agree. How would you like to be addressed? Do you have any questions beforehand? |  |
|  | **Start audio recording** We will now begin the interview. |  |
| Topics Use of Airbridge | | |
| Opening | What does your work week currently look like? |  |
| Perceived Usefulness | | |
| Worktasks | How do you use Airbridge in your daily work activities? How have your tasks as a nurse (specialist/consultant) changed since using Airbridge?  What did your work look like before using Airbridge?  Have any tasks been added or replaced due to the use of Airbridge → Which tasks?  What agreements have been made within the team regarding keeping Airbridge up to date? |  |
| Effectiveness | Do you feel that your work has become easier or more complex since using Airbridge? Could you explain this? |  |
| Topics Perceived use | | |
| Use | What can be changed to improve the use of Airbridge?  Would you recommend Airbridge to a colleague from another hospital who is not yet working with it? Could you give a score on a scale from 0–10? (0 means “definitely not”, 10 means “definitely yes”).  Can you explain why it is not a **xx** (higher score)? Can you explain why it is not a **xx** (lower score)? |  |
| Tools/applications | Which Airbridge tools do you use in your daily work? How do you experience the use of these tools?  What prevents you from using these tools? |  |
| Topics Shaping the nurse-patient relationship | | |
|  | In what way does Airbridge influence your relationship with the patient? | How do you experience this?  Do you experience any limitations? |
| Person centred care | How does Airbridge influence the way you communicate with your patients? | How do you experience this? |
|  | I would like to talk with you about the concepts of person-centred care and patient autonomy in relation to Airbridge  Can you tell me what person-centred care means to you?  In what way does Airbridge contribute to this?  In what way does Airbridge contribute to patients’ autonomy? Could you tell me more about this?  In what way does Airbridge contribute to the process of shared decision-making with patients? Could you elaborate? |  |
| Topics Commitment to the nurse-patient relationship | | |
|  | What agreements have you made with your patient regarding Airbridge?  Have there been situations in which these agreements could not be adhered to?  Are there situations in which you doubt what to do in relation to working with Airbridge?  How do you deal with situations in which a patient does not adhere to the agreed arrangements? |  |
| Closing question | Do you feel that Airbridge has added value to your work as a nurse?  Could you elaborate on this? What is the added value of Airbridge for your work? | What is the added value of Airbridge for your work as a nurse? |
|  | Are there any other issues or topics you would like to mention in relation to working with Airbridge or because of this interview? |  |
| Closing | If you are interested, I can send you a report on the study. Would you be interested in this? Yes/ No  I would like to send you the transcript of this interview so you can review it. If you have any additional information, please let me know. Responding is not obligatory. Do you agree with this? Yes/ No  If yes, to which (email) address may I send the report and/or transcript? |  |
